# Analysis of 10,000 ESTs from lymphocytes of the cynomolgus monkey to improve our understanding of its immune system

**DOI:** 10.1186/1471-2164-7-82

**Published:** 2006-04-18

**Authors:** Wei-Hua Chen, Xue-Xia Wang, Wei Lin, Xiao-Wei He, Zhen-Qiang Wu, Ying Lin, Song-Nian Hu, Xiao-Ning Wang

**Affiliations:** 1 Beijing Institute of Genomics, Chinese Academy of Sciences, Beijing, China; 2 School of Bioscience and Bioengineering, South China University of Technology, Guangzhou, China; 3 Graduate University of Chinese Academy of Sciences, Beijing, China

## Abstract

**Background:**

The cynomolgus monkey (*Macaca fascicularis*) is one of the most widely used surrogate animal models for an increasing number of human diseases and vaccines, especially immune-system-related ones. Towards a better understanding of the gene expression background upon its immunogenetics, we constructed a cDNA library from Epstein-Barr virus (EBV)-transformed B lymphocytes of a cynomolgus monkey and sequenced 10,000 randomly picked clones.

**Results:**

After processing, 8,312 high-quality expressed sequence tags (ESTs) were generated and assembled into 3,728 unigenes. Annotations of these uniquely expressed transcripts demonstrated that out of the 2,524 open reading frame (ORF) positive unigenes (mitochondrial and ribosomal sequences were not included), 98.8% shared significant similarities (E-value less than 1e-10) with the NCBI nucleotide (nt) database, while only 67.7% (E-value less than 1e-5) did so with the NCBI non-redundant protein (nr) database. Further analysis revealed that 90.0% of the unigenes that shared no similarities to the nr database could be assigned to human chromosomes, in which 75 did not match significantly to any cynomolgus monkey and human ESTs. The mapping regions to known human genes on the human genome were described in detail. The protein family and domain analysis revealed that the first, second and fourth of the most abundantly expressed protein families were all assigned to immunoglobulin and major histocompatibility complex (MHC)-related proteins. The expression profiles of these genes were compared with that of homologous genes in human blood, lymph nodes and a RAMOS cell line, which demonstrated expression changes after transformation with EBV. The degree of sequence similarity of the MHC class I and II genes to the human reference sequences was evaluated. The results indicated that class I molecules showed weak amino acid identities (<90%), while class II showed slightly higher ones.

**Conclusion:**

These results indicated that the genes expressed in the cynomolgus monkey could be used to identify novel protein-coding genes and revise those incomplete or incorrect annotations in the human genome by comparative methods, since the old world monkeys and humans share high similarities at the molecular level, especially within coding regions. The identification of multiple genes involved in the immune response, their sequence variations to the human homologues, and their responses to EBV infection could provide useful information to improve our understanding of the cynomolgus monkey immune system.

## Background

Non-human primates are ideal animal models for many human diseases because of their closely related genetic relationship and numerous biological and behavioral similarities with humans. As an important example, the cynomolgus monkey (*Macaca fascicularis*) is one of the most widely used surrogate animal models for the studies of infectious diseases, organ transplantation, productive biology, and development of new vaccines.

Beyond a few sequences of the major histocompatibility complex (MHC) classical class I and II genes and cDNAs, at present little information is available about the genomic and gene expression background of the immune system of the cynomolgus monkey. Because the cynomolgus monkey serves as an ideal animal model for *in vivo *HIV and other simian virus infections [1-5], HIV vaccine trials [6], organ transplantations [7,8], tuberculosis [9], and stress-related mood disorders in females [10], such knowledge could be critical to basic genetic and clinical studies.

Expressed sequence tag (EST) projects provide a rapid and relatively efficient method for gene discovery, especially in organisms that have little information on genomics. Another advantage of using cDNA sequencing is that gene information is subjected to comparative genetic analysis among closely related species, for example, human and chimpanzee, which could greatly facilitate the evolutionary and genetic human studies, since the old world monkeys share high similarities with humans at the molecular level, especially within coding regions.

Therefore, we adopted the EST strategy, sequenced and analyzed a collection of 8,312 ESTs from an Epstein-Barr virus (EBV) [11]-transformed B-lymphocyte cDNA library of a cynomolgus monkey. Many genes that are homologous to their human counterparts corresponding to antigen presentation, recognition and immune response, including MHC class I and II antigens and many clusters of lymphocyte differentiations, are present in our library, along with many other cDNAs. This information would provide us a better understanding of the immune system and genomic background of the cynomolgus monkey at the genomic level. Our data has been deposited in the GenBank database under accessions DW522370-DW530304.

## Results and discussion

### Library construction and cDNA sequencing

**Figure 1:**
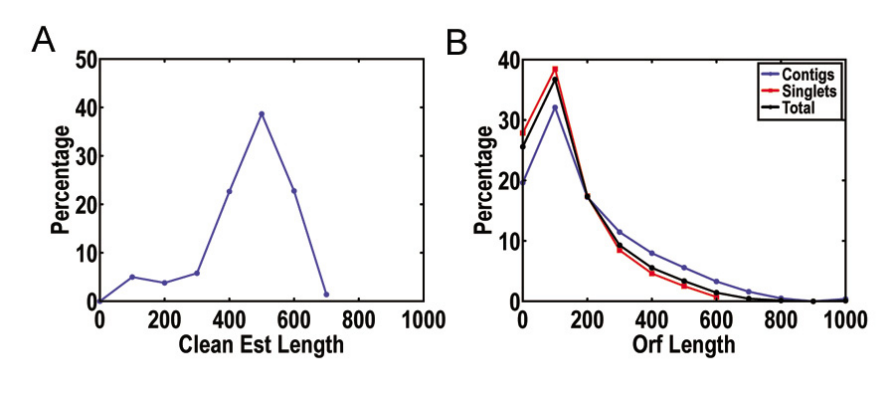
**Statistics of ESTs obtained from the cynomolgus monkey cDNA library**. The length distributions of initial ESTs (A) and putative ORFs of unigenes after assembling (B).

Lymphocyte cells were harvested and used to generate a non-normalized, directional cDNA library. Around 10,000 clones were randomly picked from the cDNA library and subjected to single-pass 5' sequencing using the T3 universal primer located at the up-stream of the vector backbone. After trimming low-quality and vector sequences and removing contaminant host sequences, a total number of 8,312 high-quality ESTs were obtained with a mean length of 509 bp. The length distributions of the ESTs are shown in Figure 1A.

### Gene-oriented and non-oriented clustering

Gene-oriented clustering in EST analysis is often applied to the species that have had much sequence information on genes and/or genomes such as human and mouse. Although we have little such information of the cynomolgus monkey, the fact that great apes and humans share 98% similarities of their coding regions indicates that it is possible to use human mRNAs as references to direct gene-oriented clustering on cynomolgus monkey ESTs.

Therefore, we downloaded all of the human mRNA reference sequences from the NCBI RefSeq database (release 14) [12] and compared them with our ESTs using BLASTN. The ESTs that aligned to the same human mRNA with E-value≤1e-6 were assembled separately along with the aligned reference sequences using the phrap program [13]. At this stage, more strict parameters (-minmatch 40 -minscore 60) of phrap were used to distinguish individual members of possible multi-member gene families. Then all assembled unique sequences and ESTs that did not have significant similarities with human mRNAs were assembled together at a low level of stringency (with default parameters). The assembled results were manually examined using CONSED [14]. Of the total 8,312 high-quality ESTs, 5,600 (67.4% of total ESTs) were assembled into 1,016 contigs, while 2,712 (32.6% of total ESTs) remained as singlets. The distributions of putative open reading frames (ORFs) of these unigenes (including contigs and singlets) are shown in Figure 1B.

**Figure 2:**
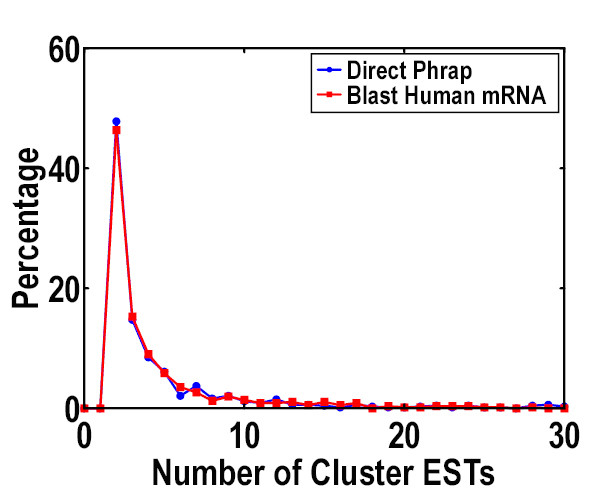
**Comparison of results of gene-oriented clustering (blast human mRNA) and non-gene-oriented clustering (direct phrap) clustering**. The two clustering methods produced similar numbers of EST clusters, the distributions of the two sets of EST clusters were also similar.

Because ESTs are single-pass sequences and are usually error prone, the introduction of reference sequences can produce more reliable results, especially for those cDNA libraries containing low-quality sequence data. We also assembled our ESTs using the original (non-oriented clustering) method and compared the results with that of gene-oriented clustering (Figure 2). It showed that the two methods produced similar results on our ESTs, indicating the high quality of our data from another point of view.

The use of the reference sequences also enabled us to identify full-length sequences or full ORFs of our total 3,728 unigenes. BLAST searches against the human mRNA reference sequences indicated 3,128 unigenes matched to the database, among which 584 (15.7% of total unigenes) extended further upstream than the ATG (methionine) start codon of their homologs, including 305 contigs and 279 singlets. 4,864 full insert sequences of cynomolgus monkey cDNAs were also downloaded from NCBI and compared with our unigenes. The results yielded additional six full-length sequences, including one contig and five singlets. This relative low percentage of full-length inserts was mainly caused by the fact that the method we used was not optimized to generate a full-insert cDNA library. A summary of the alignment results is shown in Additional file 1.

Our following analysis was based on the results of gene-oriented clustering.

### Similarities to NCBI nt and nr databases

Before the annotation process, the longest putative ORFs of all unigenes were determined by dynamically translating them in all six reading frames. Those unigenes containing too short ORFs (≤ 90 nucleotide bases or 30 amino acids) were considered non-informative ones and were subsequently excluded from the following analysis. The remaining 2,898 ORF positive unigenes (containing 7,397 ESTs) were compared with the NCBI nucleotide (nt) database and the NCBI non-redundant protein (nr) database using BLASTN and BLASTX, respectively. The searching results indicated that 183 unigenes were annotated to mitochondria encoded sequences, containing 2,768 ESTs; whereas 191 unigenes were annotated to genes coding for ribosomal proteins, containing 901 ESTs. Out of the remaining 2,524 unigenes (containing 3,728 ESTs), 2,493 (98.8%) had significant similarities (E-value≤1e-10) with sequences in the nt database, containing 3,691 (99.0%) ESTs; whereas 1,709 (67.7%) had significant similarities (E-value≤1e-5) with sequences in the nr database, containing 2,596 (69.6%) ESTs. Table 1 shows the most abundantly expressed unigenes (mitochondrial and ribosomal sequences were not included) containing at least 15 ESTs and their annotations by BLASTN and BLASTX.

The annotation rate of the unigenes by BLASTX is surprisingly lower than that by BLASTN (67.7% versus 98.8%). With careful examinations, we found that 80.2% of the annotated unigenes (1,999 out of 2,493) by BLASTN have best matches with mRNA and genomic sequences from closely related organisms, such as *Homo sapiens*, *Pongo pygmaeus*, *Pan troglodytes*, and *Macaca mulatta*, which indicated that they could be the mostly expressed genes in these primates due to their numerous biological and genetic similarities.

### Identification of new transcripts in cynomolgus monkeys and the application in comparative analysis

To identify new genes in cynomolgus monkeys, we compared the 784 unigenes that did not have significant matches to the nr database with public available 98,325 ESTs of cynomolgus monkeys downloaded from the NCBI dbEST database [15] using BLASTN. We set a cutoff for significant matches as over 100 bp coverage of unigenes and 85% identities. The BLAST results indicated that 420 unigenes were matched to the cynomolgus monkey cDNAs. Then we compared the remaining 364 unigenes with human ESTs in the NCBI dbEST database and resulted in additional 225 matches. Consequently, 139 new transcripts containing at least 90 bp ORFs were identified in total. We aligned these unigenes with the human genome sequences (build 35 finished assembly from UCSC) using BLAT [16], and found 64 unigenes could not match to the human genome with the same criteria indicated above, some of which may represent novel genes specifically expressed in cynomolgus monkeys.

For the 75 unigenes that matched to the human genome, we further analyzed their aligned regions by comparing the BLAT results with the refGene database from UCSC [17]. They fell into four categories based on the regions they aligned to (Table 2): those aligned to intergenic regions (class I), those aligned to introns of known human genes (class II), those overlapped the intron and exon regions with known genes (class III) and those aligned to untranslated regions (UTRs) (class IV), which included 21, 39, 8 and 7 unignes respectively. It is most likely that class I unigenes represent new genes in the human genome. Some of class II-IV unigenes may represent missing exons or alternatively spliced ones missed in the current human genome annotations, but it is possible that most of the class II unigenes may represent new genes, since most of which aligned to multiple blocks of the introns.

The comparative results could be very helpful for us to discover more human genes that are currently not present in the human genome annotation reservoir and to revise those incompletely or incorrectly annotated ones by using genes from cynomolgus monkeys as probes and primers. The old world monkeys are highly similar to humans at the molecular level, especially within coding regions. For example, we share over 98% of our genes with apes. By comparing the novel transcripts in cynomolgus monkeys with the human genome and mapping these transcripts to human and chimpanzee chromosomes, we could isolate new genes that may not be present in current gene annotation reservoirs of both species.

Another advantage that we can take from genes expressed in cynomolgus monkeys is to improve the overall performance of gene-finding programs by comparative methods. Currently, *de novo *methods can correctly predict only 70% of individual exons and all the coding exons of 20% genes in the human genome, with a large number of false-positives [18]. The situation for our dataset is even worse. We compared the 75 unigenes with predicted annotations of the human genome performed by genScan, twinscan, geneid, mgcGenes and sgpGene using BLAST. The results demonstrated that only two unigenes significantly matched to the genScan prediction, one to the geneid and none to the remaining three predictions. With the aids from comparative methods employing similarities among multiple genomic sequences and gene structure conservations, *ab initio *gene-finding programs could provide high enough specificities and outperform those purely gene prediction methods [19-21].

### Gene Ontology

**Figure 3:**
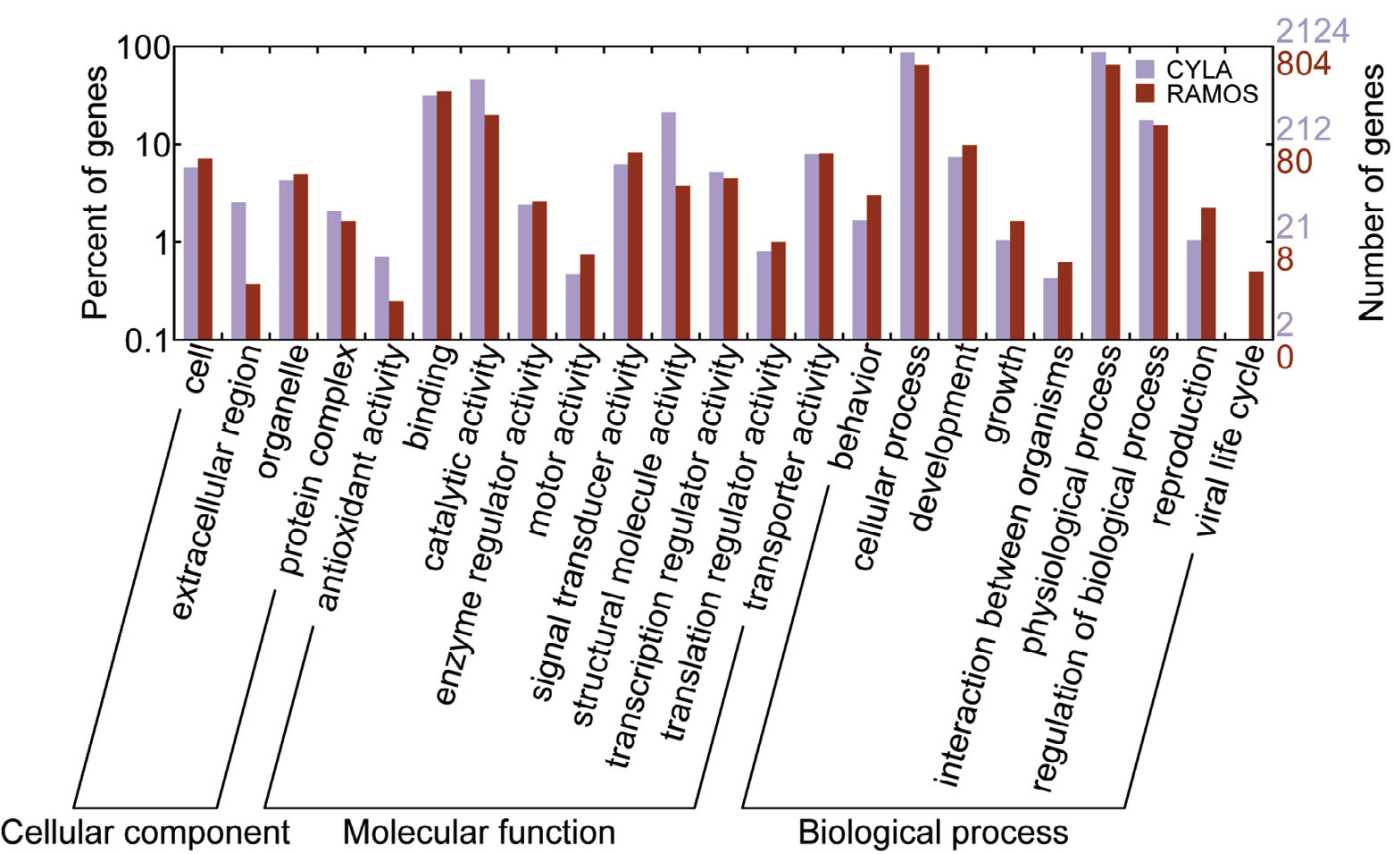
**The Gene Ontology (GO) categories of genes from the cynomolgus monkey lymphocyte cDNA library (CYLA) and the RAMOS cell line (RAMOS)**. The genes were functionally categorized according to the Gene Ontology Consortium and level two of the assignment results were plotted here. In this ontology, "biological process", "cellular component" and "molecular function" are categorized independently. 57% (2,124 of total 3,728 unigenes) unigenes from the cynomolgus monkey cDNA library and 63.9% unigenes from the RAMOS cell line were classified by GO.

Because the cDNA library was derived from transformed B cells without normalization, it represents a gene expression profile at idealistic environments. By analyzing all unigenes for their functional characteristics using the Gene Ontology (GO) [22], we can overview the gene expression profile of our cDNA library. In this ontology, we classified 2,124 (57% of total 3,728 unigenes) of our unigenes into three functional categories, "biological process", "cellular component" and "molecular process", which were treated as independent attributes. Because some genes were classified into more than one subcategory in each of the three major categories, the sum of unigenes in subcategories of every major category might exceed 100%. An overview of the classification is shown in Figure 3. The detailed GO assignment results are listed in Additional file 2.

The largest subcategory found in "biological process" was physiological process, consisting 87.4% of the unigenes in this category. The second largest subcategory was cellular process, consisting 87.0% of the unigenes. These abundantly expressed genes indicated that cells were undergoing rapid growth and metabolism, which is consistent with the materials that the cDNA library was derived from. Other highly abundant subcategories including binding and catalytic activity in "molecular function" also confirmed this conclusion.

**Figure 4:**
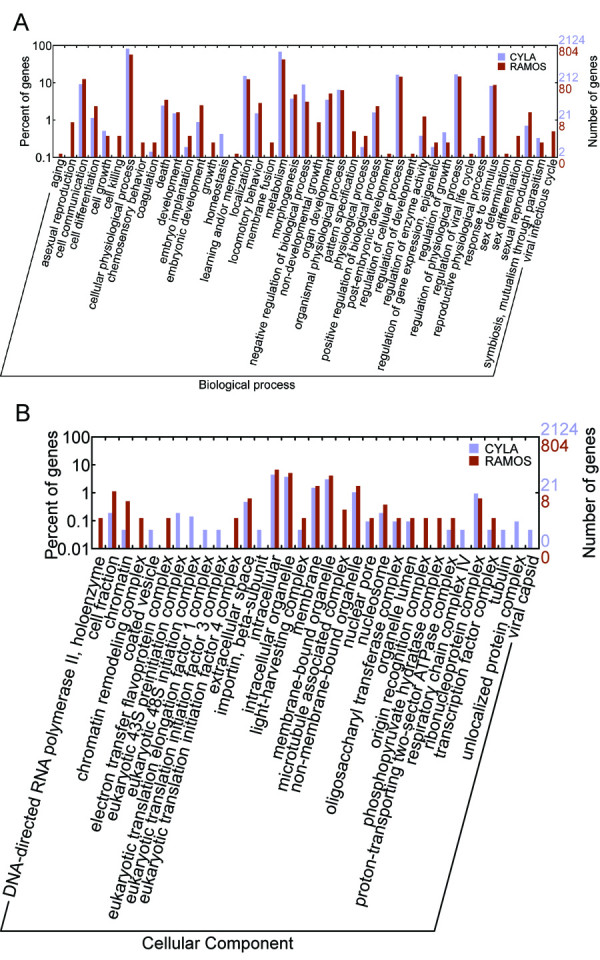
**The level 3 GO classification of genes from the cynomolgus monkey lymphocyte cDNA library (CYLA) and the RAMOS cell line (RAMOS)**. The "biological process" (A) and "molecular function" (B) are shown here.

The GO function categories for our library were further compared with a human B cell (RAMOS, a Burkitt's lymphoma derived cell line) cDNA library (dbEST Library ID.13050) downloaded from the NCBI UNIGENE database (Figure 4). The Burkitt's lymphoma (BL) is known as a tumor that is infected by EBV and consists of B lymphocytes. Total 5,374 ESTs from the non-normalized RAMOS cDNA library were assembled into 1,579 unigenes following our protocol, and 1,257 unigenes were annotated by BLASTX, in which 63.9% unigenes were classified into the GO functional categories. As a percentage of classified genes, these two cDNA libraries were similarly distributed among the growth dependent groups in both functional categories of molecular function and biological process (Figure 4), such as binding, catalytic, cellular process and physiological process. These subcategories also represented the most abundant ones in both cDNA libraries. Because B cells in both libraries were featuring rapid metabolism and unlimited cell growth, the equal distributions of these abundantly expressed groups of genes are likely responsible to their tumor-like characteristics.

Careful examinations of the genes involved in biological process of these two cDNA libraries (Figure 4A) revealed that much more genes specifically expressed in the RAMOS cell line were involved in the process of cell differentiation and non-developmental growth, such as aging, death, morphogenesis and pattern speciation, indicating the non-uniformity development of the RAMOS cells. In cellular component (Figure 4B), most of the unigenes that are RAMOS-specific were localized in complexes that associated with the formation of DNA primary packing unit into higher structures of chromosomes, which indicated that the gene transcription was mediated by selectively packing target regions on chromosomes and more transcription regulators were produced in the RAMOS cells comparing with our cDNA library. Therefore, the gene transcriptions in the RAMOS cells were biased although they were also featuring rapid growth and metabolisms. This is likely to be a result of that the RAMOS cells were of high-passage and tended to be more morphologically and genetically modified and subjected to apoptosis.

### Gene families and functional domains assignment

The latest InterPro [23] database that represents a collection of protein families, domains and functional sites in which identifiable features found in known proteins can be applied to unknown protein sequences was downloaded from the website of European Bioinformatics Institute and was compared with our unigenes using BLASTX with E-value≤1e-5. Table 3 shows the top 10 most frequently occurring protein families and functional domains assigned to our cDNA library. The largest protein family and domain was assigned to immunoglobulin/MHC, including 41 individual unigenes, followed by immunoglobulin-like, cytochrome c heme-binding site and immunoglobulin C-type, including 38, 37 and 33 unigenes, respectively. These unigenes include proteins that are responsible for the molecular basis of the blood group antigens, consisting at least two light chains: lambda and kappa, and several heavy chains of immunoglobulin, as well as MHC class I and II antigens. Other functional important immunoglobulins including a B cell differentiation antigen CD19, a TAP binding like protein and several TNFSF members are also on the list. Note that the 1^st^, 2^nd ^and 4^th ^most abundantly expressed protein families were all immunoglobulin-related, indicating that our cDNA library is suitable for the discovery of genes involving in antigen representations and recognitions, protein-protein and protein-ligand interactions, which could improve our basic understanding of the immune system of the cynomolgus monkey.

### MHC-related genes

Genetic studies have revealed that MHC associates with more human diseases than any other regions of the human genome, including most of the immune-related disorders, for example, insulin dependent diabetes mellitus (IDDM), rheumatoid arthritis (RA), ankylosing spondylitis (AS), common variable immunodeficiency (CVID) and IgA deficiency (IgAD) [24]. Strong associations have been found between many diseases and alleles of classical HLA class I and II genes. The genes within MHC class III region were also thought to be responsible to susceptibilities to many diseases [25].

The using of the cynomolgus monkey as a surrogate animal model has greatly facilitated the developments of new medicines against human diseases, especially those immune-related ones such as HIV and heart transplantation. But the lacking of knowledge on the MHC region of the cynomolgus monkey has become a bottleneck to basic genetic researches and bio-medical industries.

By constructing the cDNA library using B lymphocytes that play important roles in antigen-representation in the immune system, we expected as many as possible genes, especially MHC-related ones. In our cDNA library, totally 39 unigenes were annotated to MHC antigens using BLASTX, including four MHC class I genes: MHC_A, B, E and F, eight MHC class II molecules, and five ones homologous to HLA-related genes: HLA_B associated transcript 2 (BAT2), complement component 4B (C4B), nuclear factor of kappa light polypeptide gene enhancer in B-cells inhibitor-like (NFKBIL1), valyl-tRNA synthetase and casein kinase 2, consisting 43, 129 and 14 ESTs, respectively.

To evaluate the degree of sequence similarity of the MHC class I and II genes between cynomolgus monkeys and humans at both nucleotide and protein levels, we analyzed the overlapped sequences with the coding sequences of their corresponding human references. The overall protein similarities were slightly higher than that of nucleotides (Table 4), indicating low rates of nonsynonymous substitutions. The MHC class I genes showed weak amino acid similarity (<90%) to the human reference sequences. We were not surprised with such low sequence identities, since the class I molecules have higher polymorphisms than any others in primates. There is also evidence reported for increased MHC divergence and positive selection among members of mammals [26]. Totally 109 amino acid substitutions were identified at the four MHC class I unigenes by comparing to the human reference sequences (data not shown). 21 of these fell into the transmembrane region (exon 5) and the cytoplasmic tail (exon 6 and 7), 76 fell into the peptide binding domain (exon 2 and 3) and 12 fell into other regions. Since exon 2 and exon 3 are responsible for the peptide binding specificity of each class molecules and usually of high polymorphisms, we speculate at least part the differences and all in other regions between cynomolgus monkeys and humans are results of adaptations to their different environments. MHC class II genes showed relatively high similarity to their human counterparts. Taking two full-length inserts, DRA and DRB for example, we identified 7 and 21 amino acid substitutions, respectively. All substitutions fell into the leading peptide (exon 1) and the two extracellular domains (exon 2 and 3); none fell into the transmembrane domain (exon 4). Because these molecules show no polymorphism in the human genome, the substitutions may represent basic differences in the immune responses between cynomolgus monkeys and humans.

**Figure 5:**
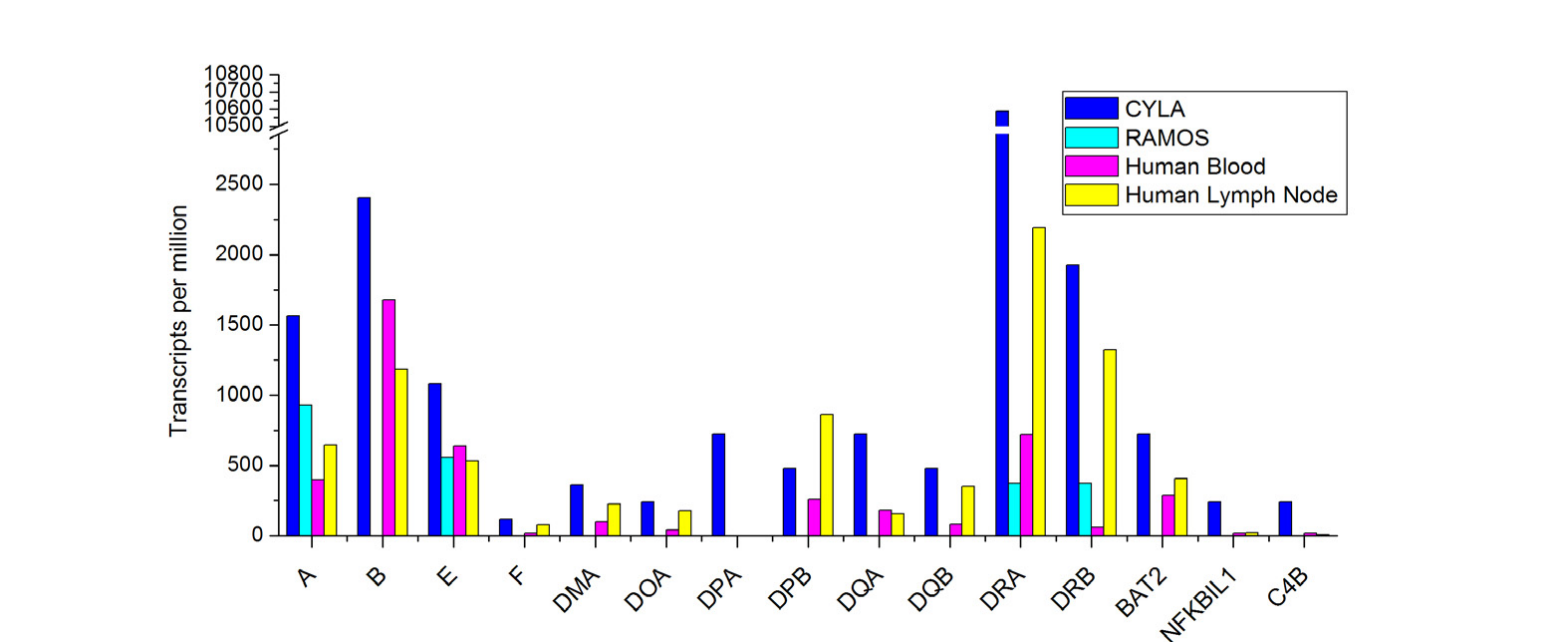
**Comparisons of gene expression profiles of MHC class I, II and III molecules**. For the comparison purpose, the expression data of MHC genes in the human blood and lymph node were downloaded from the NCBI UNIGENE database and compared with that of the RAMOS cell line and our cDNA library of cynomolgus monkey B lymphocytes (CYLA). The gene expression abundance was indicated as "transcripts per million" for the comparison convenience.

**Figure 6:**
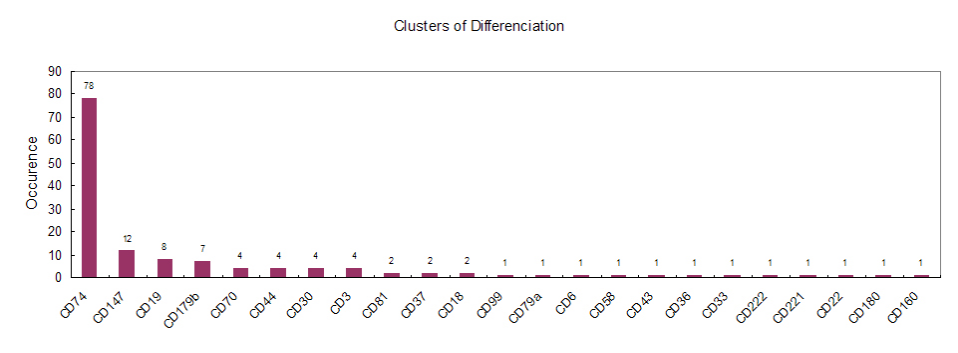
The gene expression abundance of Clusters of Differentiation of lymphocytes in our cDNA library (CYLA).

The relative expression abundances of the MHC-related genes as transcripts per million were calculated and compared with those in the RAMOS cell line and two most related human tissues to B cells: blood and lymph node (Figure 5). Since MHC molecules play important roles in concomitant recognition of tumor antigens and are critical for initiation and implementation of the cellular immune response, their abnormal expression levels are often associated with the progression of malignant transformation. Low levels of MHC expression lead to low efficiency in antigen presentation and result in boosting of the cell proliferation. In RAMOS, for example, almost all MHC molecules were down-regulated or even absent (Figure 5). But in our cDNA library, we observed that almost all MHC antigens were up-regulated, which was apparently contradictory to the theory. Interestingly, the RAMOS cell line expressed higher levels of HLA-A and HLA-E than human lymph node, suggesting a possible option for disrupting the antigen-recognition/representation pathways: selectively down-regulating key members instead of quenching all of which in malignantly transformed cells. For example, a functional heterodimer DM, which is composed of two subunits MHC-DMA and DMB, is required for MHC class II/peptide complex formation in antigen-presenting cells [27]. Although the DMA molecule was up-regulated in our cDNA library, the absence of the DMB molecule indicated that the proper functions of B cells might be interrupted. However, the situation for the DR heterodimer that plays a central role in antigen representation is different, since the expression levels for both of its subunits DR alpha and beta were up-regulated comparing with that in the human blood and lymph node. Interestingly, the MHC class II antigen DR alpha was also found over-expressed in many types of cancers. Although not in common, DR beta did simultaneously express with DR alpha in many cancer types [28], suggesting the level of DR beta transcripts was regulated by a post-transcriptional or post-translational mechanism. In addition, high levels of the invariant chain (Ii/CD74, see Figure 6) that is essential to antigen presentation were observed in our cDNA library, indicating that some key member(s) were lacking of mature protein expression, which provides the third optional way to interfere the host immune system.

### Lymphocyte differentiation antigens, related ligands and receptor genes

Lymphocytes can be divided into subsets either by their functions or by surface markers that have been designated as Clusters of Differentiation (CDs), which play various but central roles in B cell activation, development and immunological response regulation. Currently, as many as 356 CDs [29] have been identified, although many of which were not functionally assigned. Here we present 24 CDs identified in our library and their expression abundances (Figure 6), among which CD19, CD22, CD37, CD74, CD79a, CD81, CD179a and CD180 are B-cell associated surface markers.

CD19 is a B-cell specific molecule that regulate B cell development, activation, differentiation and antigen receptor-mediated MHC class II antigen processing [30]. The membrane complex formed by CD19/CD21/CD81 serves as the co-receptor for B-cell receptor (BCR) and lowers the thresholds for B cell activation [31,32]. CD21 (complement receptor 2, CR2) serves as the EBV receptor of human B lymphocytes [33]. A lack of this EBV receptor on transformed B cells was described as a reason for long-term EBV seronegativity [34], which explains the absence of CD21 from our cDNA library.

CD74 is the most abundant one among the surface markers, which contained 78 ESTs (9,834 transcripts per million ESTs), comparing with 11,533 transcripts per million ESTs in the human lymph node. CD74 plays an important role in the antigen representation by acting as a specific chaperone in MHC class II assembly and transport [35] and was found coordinately expressed with the MHC class II antigens in human fetal tissues [36]. Recent studies have shown that CD74 is a membrane receptor for the macrophage migration inhibitory factor (MIF, containing 19 ESTs), and is responsible for the proliferation by activating the mitogen-activated protein kinase (MAPK) pathway [37] and cytokine expression such as tumor necrosis factor (TNF)-alpha and interleukin (IL)-beta in cancer cells [38]. CD44 (containing four ESTs) that functions in cell proliferation and adhesion is another receptor of MIF. A TNF super family member 7 (CD70, containing four ESTs) and a TNF receptor super family member 8 (CD30, containing four ESTs) were expressed in our cDNA library, constituting an activation pathway for B cell proliferation along with the MIF-CD74 plus CD44 complex.

After infection by EBV, B cells were immortalized and some tumor-like features would appear. Several CD members indicated such state of abnormity. For example, CD147 (basigin; EMMPRIN), the second most abundant CD in our cDNA library, is a major mediator of malignant cell behavior in the human, and is regulated by several membrane-associated cofactors, including annexin II (containing one EST) and caveolin-1 [39]. These surface makers could be used as targets of new medicines against tumor growth. CD179b that only exists in proB and preB cells involving in cell proliferation and differentiation from the proB to preB cell stage was also expressed in our cDNA library, indicating the non-differentiation stage of the transformed B cells. Strangely, some T-cell specific surface markers also expressed in our cDNA library, such as CD3 and CD160 (Figure 6), indicating possible contaminations from T-cell or other cell types while transformed by EBV.

### Other immunoglobulin genes

Besides MHC genes and many CDs, several other immunoglobulin genes were identified in our library, including immunoglobulin alpha heavy chain constant region (C alpha), immunoglobulin kappa-chain and immunoglobulin lambda-chain. Recent studies showed that alleles of a single immunoglobulin C alpha chain were presented in rhesus macaques (*Macaca mulatta*) [40], indicating that similar intra-species heterogeneity of the immunoglobulin genes may exist in cynomolgus monkeys. Since these molecules represent the major antibody class functioning as a first line of defenses by neutralizing invading pathogens, their polymorphisms suggest that possible differences among individual animals should be taken account when designing experiment strategies to induce antibodies.

Although with the absence of some key molecules in the activation of immune responses to facilitate the virus transformation, the transformed B lymphocytes from the cynomolgus monkey expressed more immunoglobulin and ligand and receptor genes than in human blood and lymph nodes, in numbers as well as in abundances; A B-cell specific proliferation pathway including MIF, CD74 and CD44 was greatly enhanced after transformation. These results indicated that this cDNA library represents an ideal model to improve our understanding of mechanisms of antigen representations and immune responses of cynomolgus monkeys. The differentially expressed genes between our cDNA library and normal B cells of cynomolgus monkeys, if available, should be in great interests, because of their possible key functions corresponding to the immune response and malignant cell growth. Yet one of the greatest challenges is that we do not know which one of the differentially expressed genes is the reason of the transformation and which one is just the result. Much more researches are needed to be done until we get the answer.

## Conclusion

We have constructed a cDNA library from lymphocytes of the cynomolgus monkey and sequenced about 10,000 randomly picked clones. Approximately all (98.8%) ORF positive unique transcripts (mitochondrial and ribosomal sequences were not included) shared significant similarities with the NCBI nt database, but only 67.7% of these transcripts shared significant similarities with the NCBI nr database using BLAST-based tools. Further analysis revealed 420 new transcripts in cynomolgus monkeys; in which 139 did not share significant similarities with any human ESTs. Such sequence information was used in comparative analysis to identify possible novel genes in the human genome.

The cynomolgus monkey is one of the most widely used surrogate animal models for an increasing number of human diseases and vaccines, such as HIV and heart transplantation. But the lacking of background information at the genomic level especially in the immune system has greatly hinged our progress. Our cDNA library, which was derived from the EBV-transformed B lymphocytes of the cynomolgus monkey, could identify as many as possible immune-related genes and reveal the gene expression profile. Since many human diseases are associated with the MHC region and deficiencies of immune system, such knowledge is of great interest to researchers and industries that use cynomolgus monkeys as experimental models.

## Methods

### *In vitro *transformation of cynomolgus monkey B lymphocytes by EBV

An amount of 4–5 mL whole blood was sampled from a cynomolgus monkey, followed by the isolation of lymphocyte cells. The B-lymphocyte cell line was established according to Scammell *et al *[41].

### Construction and quality assessment of the cynomolgus monkey cDNA library

Total RNA was extracted from culturing cells using the Trizol agent (Invitrogen) according to the manufacturer's instruction. mRNA was subsequently purified using the mRNA PolyATtract@mRNA isolation system (Promega). The cDNA library was constructed in the directional pBluescript^® ^II XR vector (Stratagene) exploiting the EcoRI and XhoI restriction sites according to the manufacturer's instruction.

The quality of the cDNA library was first assessed by colony PCR of 96 randomly picked clones to determine the average insert size and percentage of clones without inserts. Then 384 randomly picked clones were sequenced to determine the ratio of contaminations (vector, *E. coli*) and valid average (masked) length. The randomicity of the cDNA library was determined by calculating the ratio of unique sequences (contigs + singletons)/reads.

### Plasmid preparation and 5'EST sequencing

Plasmid constructs were transformed into *E. coli *DH10B (Invitrogen), grown overnight on solid LB medium containing IPTG and X-Gal. 10,000 colonies were picked, grown overnight in LB medium containing Amp in 96-well plates. An amount of 5 μL from each well were transferred into 384-well microtitter plates containing 5 μL 20% glycerol (V/V) to preserve a permanent clone stock. Plasmids were isolated and sequenced on MegaBase^® ^1000 sequencers using the T3 universal primer that anneals to the plasmid backbone upstream of the 5' end of the cDNA inserts.

### Gene-oriented clustering of the cDNA sequences

Phred [42,43] was used with default parameters to determine each base call from files extracted from sequencers and reject sequences that were of low quality. Crossmatch program [13] was run to trim vector sequences. Acceptable results (e.g. >100 bp long) were saved in a FASTA format.

Human mRNA sequences from the NCBI RefSeq database were downloaded and used as reference sequences to direct clustering. Firstly, the reference sequences were compared with our ESTs using BLASTN with an E-value≤1e-6. ESTs that aligned to the same mRNA were assembled along with the mRNA using the phrap program with more strict parameters (-minmatch 40 -minscore 60) to distinguish possible individual members of possible multi-gene families.

### Sequence-similarity searches and functional class annotation

The lymphocyte unigenes were compared with the NCBI nt database using the BLASTN program and with the protein sequences in the NCBI nr database using the BLASTX program. They were also compared with public available full-length inserts as well as cynomolgus monkey and human ESTs. The E-value cutoffs were 1e-10 for BLASTN and 1e-5 for BLASTX, respectively. Functional classes were assigned according to GenBank to GO mapping provided by the GO website. Then the distributions of the gene families and functional domains were assigned according to the sequence similarities of our unigenes to the InterPro database.

## Authors' contributions

SNH and XNW participated in designing the research; XXW, XWH, ZQW, WL and YL performed the research; WHC controlled and analyzed the data; WHC and XXW wrote the paper.

## Supplementary Material

Additional File 1 **Statistics of BLAST searches against the human reference sequences (RefSeq). **The coverage was calculated by dividing the length of HSPs (high scoring pairs) by the CDS length of aligned references. Out of the 3,728 unigenes, 3,128 matched to the database. Click here for file

Additional File 2 **The GO assignment results of the cynomolgus cDNA library.** Click here for file

## Figures and Tables

**Table 1:** The most abundantly expressed unigenes containing at least 15 ESTs and their annotations in the cDNA library of the cynomolgus monkey B lymphocytes.

unigene ID	Blastx NCBI nr		Blastn NCBI nt		clustered ESTs
		
	E-value	Annotation	E-value	Annotation	
Contig998	1.00E-119	HLA-DR-gamma [Pan troglodytes]	0	Pongo pygmaeus mRNA; cDNA DKFZp469K1522	73
Contig1006	1.00E-103	Unknown (protein for MGC:22645) [Homo sapiens]	0	Homo sapiens cDNA clone MGC:22645 IMAGE:4700961, complete cds	61
Contig1003	0	glyceraldehyde-3-phosphate dehydrogenase [Homo sapiens]	0	Homo sapiens cDNA clone MGC:20338 IMAGE:4541305, complete cds	52
Contig997	9.00E-93	Chain C, Antibody Gnc92h2 Bound To Ligand	0	Macaca fascicularis CDR1, CDR2, CDR3 mRNA	36
Contig988	0	cytoskeletal beta actin [Sus scrofa]	0	Pan troglodytes actb mRNA for beta-actin, complete cds	30
Contig989	2.00E-25	TMSB4L [Homo sapiens]	0	Homo sapiens thymosin-like 3 (TMSL3), mRNA	30
Contig984	0	elongation factor 1 alpha [Canis familiaris]	0	Pan troglodytes chromosome 7 clone RP43-5L2, complete sequence	24
Contig978	5.00E-99	peptidylprolyl isomerase A isoform 1 [Pan troglodytes]	0	Pan troglodytes similar to peptidylprolyl isomerase A isoform 1	21
Contig975	1.00E-61	macrophage migration inhibitory factor [Macaca mulatta]	0	Homo sapiens macrophage migration inhibitory factor, complete cds	19
Contig971	1.00E-180	cytosolic thyroid hormone-binding protein (EC 2.7.1.40)	0	Homo sapiens pyruvate kinase, muscle, mRNA	18
Contig966	4.00E-48	Very hypothetical protein	0	Homo sapiens cDNA clone IMAGE:4566256	16
Contig961	7.00E-38	Alu subfamily SB sequence contamination warning entry	1.00E-174	Human DNA sequence from clone RP11-535C21 on chromosome 9	15
Contig964	1.00E-144	hypothetical protein [Homo sapiens]	0	Homo sapiens enolase 1, (alpha), mRNA, partial cds	15

**Table 2:** The mapping results of the 75 unigenes to the human genome.

GenBank Acc ^a^	Length	Matched length	Query Start	Query End	Chr #	Strand	# of Blocks	Aligned Position ^b^
DW529317	708	632	10	705	21	+	4	INTERGENIC
DW529878	646	574	23	646	16	+	6	INTERGENIC
DW526085	647	561	7	647	11	-	7	INTERGENIC
DW529759	590	552	1	590	14	+	4	INTERGENIC
DW527754	600	549	13	600	12	+	4	INTERGENIC
DW524476	589	549	6	589	13	-	3	INTERGENIC
DW523790	595	548	8	595	7	-	6	INTERGENIC
DW525277	592	506	4	588	7	+	4	INTERGENIC
DW529009	559	503	20	559	7	+	5	INTERGENIC
DW528198	558	479	33	554	11	-	7	INTERGENIC
DW526469	506	477	4	506	8	+	3	INTERGENIC
DW527916	554	471	39	553	17	+	9	INTERGENIC
DW529398	497	451	15	497	6	-	6	INTERGENIC
DW529781	535	447	18	535	15	+	6	INTERGENIC
DW525705	502	430	26	502	20	+	8	INTERGENIC
DW524254	496	425	47	495	18	-	3	INTERGENIC
DW522689	454	395	34	446	12	+	6	INTERGENIC
DW525332	373	328	3	373	X	+	7	INTERGENIC
DW524885	295	273	0	295	10	+	3	INTERGENIC
DW530192	546	229	111	361	17	-	3	INTERGENIC
DW527544	465	125	143	272	24	-	3	INTERGENIC
DW523170	686	619	6	686	10	+	5	INTRON
DW523214	667	603	5	667	16	+	5	INTRON
DW528007	647	574	38	647	9	+	1	INTRON
DW523012	609	571	4	609	15	-	2	INTRON
DW529881	609	565	7	609	18	+	11	INTRON
DW530259	638	565	15	638	21	-	6	INTRON
DW523678	583	563	2	583	23	-	4	INTRON
DW527753	652	538	24	635	7	-	7	INTRON
DW528094	599	536	25	599	8	+	4	INTRON
DW527168	607	529	7	594	11	-	7	INTRON
DW522397	575	513	0	561	12	-	6	INTRON
DW524969	620	511	7	620	7	+	7	INTRON
DW526445	561	505	11	561	11	-	10	INTRON
DW527944	601	505	45	601	15	+	12	INTRON
DW528528	617	504	11	577	10	+	10	INTRON
DW526355	555	498	19	555	14	-	5	INTRON
DW525206	582	495	57	582	16	-	3	INTRON
DW529061	527	478	3	527	9	+	5	INTRON
DW526882	505	474	1	505	7	-	4	INTRON
DW528258	504	466	17	504	15	+	4	INTRON
DW526174	529	455	45	529	19	+	6	INTRON
DW526535	562	448	61	562	12	+	6	INTRON
DW523662	501	438	28	501	22	-	8	INTRON
DW523912	508	434	19	508	11	-	8	INTRON
DW522996	445	406	4	445	16	+	5	INTRON
DW523702	457	403	21	457	11	+	4	INTRON
DW526118	435	400	0	427	16	-	8	INTRON
DW526200	416	370	4	416	13	-	9	INTRON
DW526109	405	361	4	405	7	+	6	INTRON
DW529322	537	325	14	537	19	-	9	INTRON
DW524008	314	288	4	314	13	-	4	INTRON
DW522817	317	287	7	317	10	+	2	INTRON
DW529594	314	261	40	314	8	-	2	INTRON
DW528820	558	255	3	293	19	-	3	INTRON
DW527252	363	233	108	355	17	-	4	INTRON
DW524169	213	198	4	213	14	-	3	INTRON
DW523610	220	195	0	213	7	-	5	INTRON
DW525236	208	132	23	169	17	+	2	INTRON
DW528483	633	122	361	566	17	-	6	INTRON
DW529395	645	565	38	637	19	+	3	OVERLAPED
DW523821	616	550	6	616	12	+	4	OVERLAPED
DW528834	552	449	43	552	11	+	9	OVERLAPED
DW523419	472	429	5	471	15	-	1	OVERLAPED
DW524249	504	173	25	216	22	-	8	OVERLAPED
DW526968	577	144	78	244	16	+	7	OVERLAPED
DW529339	605	112	3	605	X	-	7	OVERLAPED
DW527303	607	569	4	607	11	+	3	3'UTR
DW522948	155	138	6	155	7	-	4	3'UTR
DW525388	608	571	0	608	21	-	4	5'UTR
DW529480	573	544	9	573	8	+	1	5'UTR
DW527696	589	535	25	589	12	-	5	5'UTR
DW525894	610	509	20	607	21	+	6	5'UTR
DW530217	483	433	5	475	14	+	8	5'UTR
DW530077	519	352	8	381	8	-	7	5'UTR

a. The GenBank identifiers for the 75 unigenes that could be mapped to the human genome and show no homology to any sequences in the nr database and public available cynomolgus monkey and human ESTs.

b. The aligned regions were determined by comparing the BLAT results with the refGene database.

**Table 3:** The most frequently occurring protein families and functional domains in the cynomogus monkey cDNA library.

**IPR Number**	**Annotation**	**# Of Unigenes**
IPR003006	Immunoglobulin/major histocompatibility complex	41
IPR007110	Immunoglobulin-like	38
IPR000345	Cytochrome c heme-binding site	37
IPR003597	Immunoglobulin C-type	33
IPR000504	RNA-binding region RNP-1 (RNA recognition motif)	26
IPR002218	Glucose-inhibited division protein A	26
IPR006209	EGF-like domain	22
IPR007087	Zn-finger, C2H2 type	16
IPR000298	Cytochrome c oxidase, subunit III	15
IPR002429	Cytochrome c oxidase, subunit II	15

**Table 4:** Sequence similarity of MHC genes between cynomolgus monkeys and humans.

MHC gene		RefSeq ID ^a^	Nucleotide identity (%) ^b^	Amino acid identity (%) ^c^	Aligned exons ^d^
Class I	A	NM_002116	84.7	88.6	3–5
	B	NM_005514	86.1	86.5	1–7
	E	NM_005516	80.1	84.3	2–4
	F	NM_018950	73.9	75	4–6
Class II	DMA	NM_006120	94	96.6	2–3
	DOA	NM_002119	92	95	3–4
	DPA	NM_033554	93	96	2–5
	DQA	NM_002122	91.5	93	1–3
	DQB	NM_002123	94.6	95	2–3
	DRA	NM_019111	94	96	1–4
	DRB	NM_002124	88	89	1–4

a. GenBank identifiers for the human reference sequences

b. Nucleotide sequence identity between cynomolgus monkeys and humans

c. Amino acid sequence identity between cynomolgus monkeys and humans

d. Exons that MHC genes in our library aligned to the human reference sequences
