# Supplementary material for: Analysis of 10,000 ESTs from lymphocytes of the cynomolgus monkey to improve our understanding of its immune system
Source: BMC Genomics. 2006 Apr 18;7:82. doi: 10.1186/1471-2164-7-82 (PMC1522023; doi:10.1186/1471-2164-7-82)

### Additional file 1:

**Statistics of BLAST searches against the human reference sequences (RefSeq).** The coverage was calculated by dividing the length of HSPs (high scoring pairs) by the CDS length of aligned references. Out of the 3,728 unigenes, 3,128 were matched to the database.


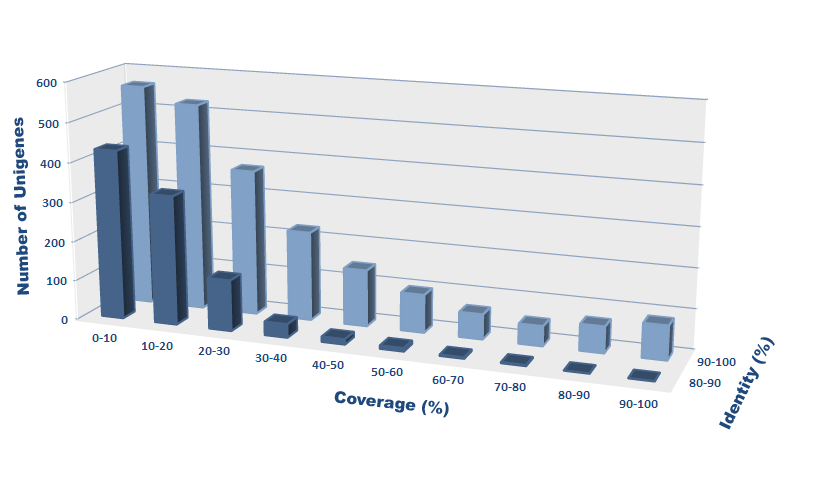

Supplement: Additional File 1 — Statistics of BLAST searches against the human reference sequences (RefSeq). The coverage was calculated by dividing the length of HSPs (high scoring pairs) by the CDS length of aligned references. Out of the 3,728 unigenes, 3,128 matched to the database. [file 1471-2164-7-82-S1.doc]
