# Supplementary material for: Analysis of 10,000 ESTs from lymphocytes of the cynomolgus monkey to improve our understanding of its immune system
Source: BMC Genomics. 2006 Apr 18;7:82. doi: 10.1186/1471-2164-7-82 (PMC1522023; doi:10.1186/1471-2164-7-82)
Supplement: Additional File 2 — The GO assignment results of the cynomolgus cDNA library. [file 1471-2164-7-82-S2.doc]

### Additional file 2:

The GO assignment results of the cynomolgus cDNA library.

| GO level 1 | GO level 2 | # of Unigene | % of Unigene ^a^ |
| --- | --- | --- | --- |
| Cellular Component | cell | 122 | 5.7439 |
|  | extracellular region | 54 | 2.5424 |
|  | organelle | 91 | 4.2844 |
|  | protein complex | 44 | 2.0716 |
| Molecular Function | antioxidant activity | 15 | 0.7062 |
|  | binding | 667 | 31.4030 |
|  | catalytic activity | 979 | 46.0923 |
|  | enzyme regulator activity | 51 | 2.4011 |
|  | motor activity | 10 | 0.4708 |
|  | signal transducer activity | 133 | 6.2618 |
|  | structural molecule activity | 447 | 21.0452 |
|  | transcription regulator activity | 110 | 5.1789 |
|  | translation regulator activity | 17 | 0.8004 |
|  | transporter activity | 169 | 7.9567 |
| Biological Process | behavior | 35 | 1.6478 |
|  | cellular process | 1847 | 86.9586 |
|  | development | 156 | 7.3446 |
|  | growth | 22 | 1.0358 |
|  | interaction between organisms | 9 | 0.4237 |
|  | physiological process | 1856 | 87.3823 |
|  | regulation of biological process | 371 | 17.4670 |
|  | reproduction | 22 | 1.0358 |
|  | viral life cycle | 2 | 0.1000 |

a. 2,124 out of total 3,728 unigenes were classified by GO.
